# The shape of fluvial bedload gravels: A large, high-quality dataset of active-channel deposits

**DOI:** 10.1016/j.dib.2022.108028

**Published:** 2022-03-06

**Authors:** Zelina Z. Ibrahim, S.J. Gale

**Affiliations:** a Faculty of Forestry and Environment, Universiti Putra Malaysia, 43400 UPM Serdang, Malaysia; b Department of Archaeology, The University of Sydney, Sydney, New South Wales 2006, Australia

**Keywords:** Fluvial bedload, Gravel shape, Modified Wentworth Roundness, Maximum Projection Sphericity, Oblate-Prolate Index, Sabeto River, Fiji

## Abstract

Isotropic bedload gravels from an active fluvial system were collected from seven stations along the length of the Sabeto River of western Viti Levu, Fiji. Sampling was confined to clasts of Navilawa Monzonite, an intrusive rock that crops out only along the upper reaches of the river. The sampled gravels consisted of stream-bed surface material obtained from transects normal to the active channel. An additional sample was collected from an outcrop of fresh Navilawa Monzonite undergoing active physical breakdown on the side of the bedrock channel immediately adjacent to the river. A total of 883 clasts, ranging in diameter (*b*-axis) from 12 to 337 mm, was collected. The long (*a*), intermediate (*b*) and short (*c*) axis of each clast was measured, along with the diameter of the sharpest corner of the maximum projection outline (*D_i_*) and the diameter of the maximum inscribed circle (*D_k_*). At six of the stations, the mass of each clast was recorded. Measurements were also made of the density of fresh Navilawa Monzonite. The dataset includes measurements of Navilawa Monzonite density and determinations of the Modified Wentworth Roundness, Maximum Projection Sphericity and Oblate–Prolate Index of each clast. At six of the stations the volume of each particle was estimated using measurements of particle mass and rock density. The repository, in Mendeley Data [1], provides a large, high-quality dataset of the shape of isotropic bedload gravels from an active fluvial system, affording information on the downstream evolution of particle shape. The dataset will be useful for sedimentologists, fluvial geomorphologists, hydraulic engineers and those concerned with fluvial bedload transport.

## Specifications Table

**Table:** 

Subject	Earth-Surface Processes
Specific subject area	Fluvial geomorphology, Fluvial sedimentology
Type of data	Table Figure Chart
How data were acquired	Samples of fluvial bedload were collected from seven locations along the length of the Sabeto River of western Viti Levu, Fiji. The samples were confined to gravels of Navilawa Monzonite, an isotropic lithology that crops out only in the upper reaches of the system. One of the sampling sites was located above the downstream boundary of the monzonite outcrop, the rest below. A sample of gravels of fresh Navilawa Monzonite undergoing active physical breakdown was collected to provide information on the shape of the particles prior to their introduction to the fluvial system. Samples of Navilawa Monzonite were obtained for the determination of rock density.
Data format	Raw Analysed Filtered
Description of data collection	The active channel was defined as comprising the unvegetated part of the cross-section. At each sampling station, a transect was established normal to the channel and across its entire width. Every gravel of Navilawa Monzonite ≳ 10 mm lying on the stream bed beneath the transect line was sampled. Samples of gravels of fresh Navilawa Monzonite undergoing active physical breakdown were taken from the edge of the bedrock channel at Station 0. The dataset comprises the following measured properties: the long (*a*), intermediate (*b*) and short (*c*) axis of each clast, along with the diameter of the sharpest corner of the maximum projection outline (*D_i_*) and the diameter of the maximum inscribed circle (*D_k_*). This information was used to calculate the Modified Wentworth Roundness, Maximum Projection Sphericity and Oblate–Prolate Index of each clast. At six of the stations, the mass of each clast was recorded. Measurements were also made of the density of the fresh Navilawa Monzonite. The particle mass and density were used to estimate particle volume.
Data source location	Geographic Entity: Sabeto River, western Viti Levu Town/Region: Nalotawa District and Sabeto District, Ba Province Country: Fiji Latitude and longitude of collected samples/data: Bounding box coordinates of 17°44′00.00″S, 177°31′00.00″E (southwest corner) and 17°40′45.00″S, 177°35′30.00″E (northeast corner).
Data accessibility	Repository name: Mendeley Data, V1 Data identification number: 10.17632/gwnfv4c6b5.1 Direct URL to data: https://data.mendeley.com/datasets/gwnfv4c6b5/1
Related research article	S.J. Gale, The shape of fluvial gravels: insights from Fiji's Sabeto River, Geosciences, 11 (2021) 161. https://doi.org/10.3390/geosciences11040161

## Value of the Data

• The dataset consists of a set of measurements of the shape of bedload particles of a single isotropic lithology taken at intervals along an active, gravel-transporting river. As the source of the gravels is restricted to a single rock type that outcrops in the headwaters of the system, the data offer the opportunity to track the evolution of particle shape downstream without the complications that might arise from the continuous addition of new material to the system.
• This dataset will benefit fluvial geomorphologists, sedimentologists, hydraulic engineers and those concerned with fluvial bedload transport.
• The data allow us to assess (i) the role that particle shape plays in sediment entrainment and differential movement, (ii) the relative importance of shape sorting and abrasion in the development of the shape of fluvial gravels and (iii) whether particle shape retains information on sedimentary processes.
• The data may be used to assess the thesis that particle shape is environmentally diagnostic. Gravel shape may also throw light on fluvial sedimentary processes, particularly the role of sorting and attrition in the development of particle assemblages. The information may be of value for assessing and calibrating laboratory experiments on attrition, downstream fining and downstream change in shape.

## 1 Data Description

**Fig. 1:**
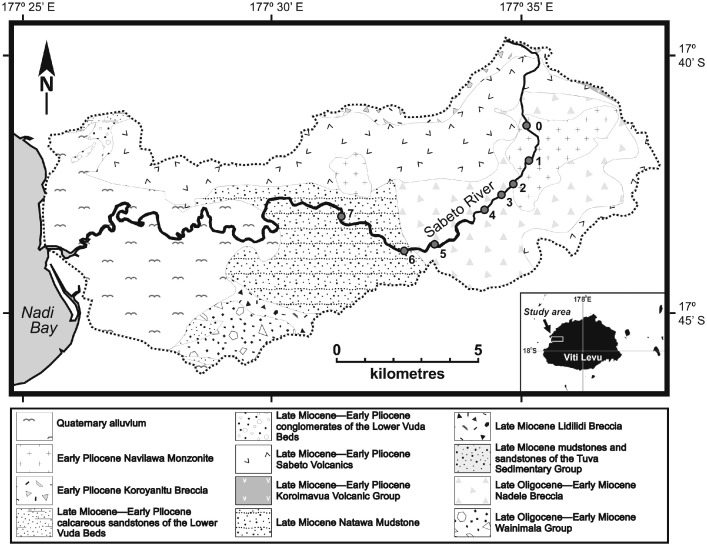
The geology of the catchment of the Sabeto River of western Viti Levu, Fiji, showing the location of the sampling stations. Modified from Gale et al [3].

This article presents a dataset of bedload gravel shape sampled from seven (7) cross-sections established at intervals along the Sabeto River of western Viti Levu, Fiji (Fig. 1). The gravels sampled are restricted to a single rock type, the Navilawa Monzonite, that crops out only in the headwaters of the system. The dataset is deposited in Mendeley Data [1]. The repository contains two files: a zipped Keyhole Markup Language (KMZ) file of the Sabeto River catchment area, geology and sampling station location, viewable with Google Earth or other compatible application; and an XLSX file of measurements of particle dimensions and derived measures of particle shape. The structure and content of both repository files are given here.

**Fig. 2:**
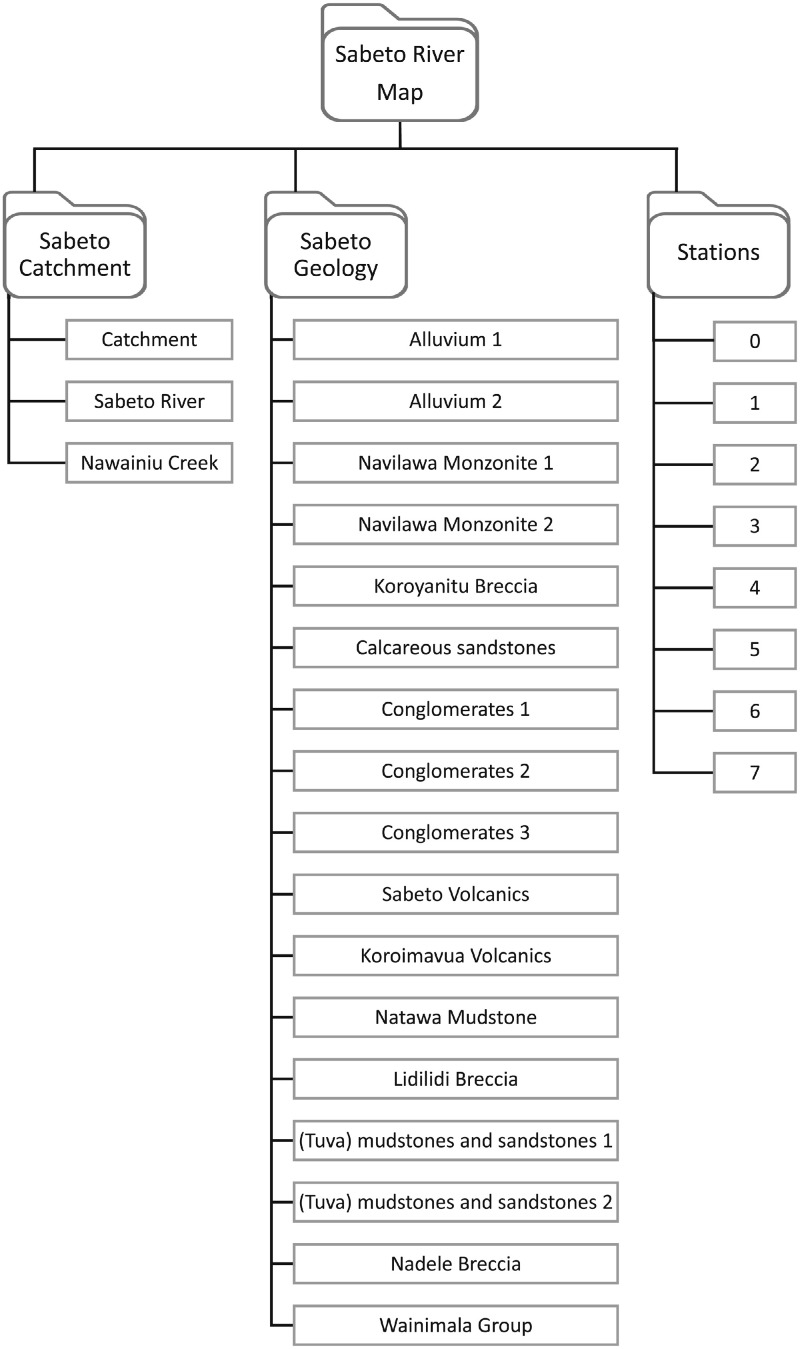
Structure and content of the KMZ file "SabetoRiverMap.kmz" in Mendeley Data [1].

The structure of the KMZ file is presented in Fig. 2. The Sabeto River catchment boundary [2] is rendered as a polygon and the main rivers of interest are displayed as paths. The file contains a map of the sampled stations as placemarks. The geological units within the catchment of the Sabeto River are presented as polygons.

The XLSX file consists of 11 worksheets (Table 1). These contain measurements of particle long (*a*), intermediate (*b*) and short (*c*) axes (mm); the diameter of the maximum inscribed circle of the particle (*D_i_*) (mm) and that of the sharpest corner on the maximum projection outline (*D_k_*) (mm); and particle mass (g). The worksheets also include the derived measures of Modified Wentworth Roundness, Maximum Projection Sphericity and Oblate–Prolate Index, estimates of particle volume (cm^3^), and the calculations involved in the estimation of the density of fresh Navilawa Monzonite.

The location of the eight stations on the Sabeto River is given in Table 2, together with the river distance [2] of the stations from the downstream limit of the Navilawa Monzonite outcrop.

Table 3 provides descriptive statistics of the range of measured values of the particle long (*a*), intermediate (*b*) and short (*c*) axes, the diameter of the maximum inscribed circle of the particle (*D_i_*), the diameter of the sharpest corner on the maximum projection outline (*D_k_*) and the particle mass.

Table 4 provides descriptive statistics of the range of calculated values of Modified Wentworth Roundness, Maximum Projection Sphericity, Oblate–Prolate Index and estimated clast volume.

**Table 1:** Content of XLSX file ``SabetoRiverMonzoniteBedloadShapeDataset.xlsx'' in Mendeley Data [1].

No.	Worksheet Name	Description
1	Contents	Short description of each worksheet
2	Station Data Summary	For each station, the following properties are listed: station location (latitude and longitude); river distance from the downstream limit of the Navilawa Monzonite outcrop; number of clasts measured; range of values of *a*-axis (mm), *b*-axis (mm), *c*-axis (mm), *D_i_* (mm), *D_k_* (mm), mass (g), Modified Wentworth Roundness, Maximum Projection Sphericity, Oblate–Prolate Index and volume (cm^3^).
3	Station 0	Measurements of *a*-axis (mm), *b*-axis (mm), *c*-axis (mm), *D_i_* (mm) and *D_k_* (mm), and calculated values of Modified Wentworth Roundness, Maximum Projection Sphericity and Oblate–Prolate Index for 23 particles.
4	Station 1	Measurements of *a*-axis (mm), *b*-axis (mm), *c*-axis (mm), *D_i_* (mm) and *D_k_* (mm), and calculated values of Modified Wentworth Roundness, Maximum Projection Sphericity and Oblate–Prolate Index for 130 particles.
5	Station 2	Measurements of *a*-axis (mm), *b*-axis (mm), *c*-axis (mm), *D_i_* (mm) and *D_k_* (mm), and calculated values of Modified Wentworth Roundness, Maximum Projection Sphericity and Oblate–Prolate Index for 125 particles.
6	Station 3	Measurements of *a*-axis (mm), *b*-axis (mm), *c*-axis (mm), *D_i_* (mm) and *D_k_* (mm), and calculated values of Modified Wentworth Roundness, Maximum Projection Sphericity and Oblate–Prolate Index for 109 particles.
7	Station 4	Measurements of *a*-axis (mm), *b*-axis (mm), *c*-axis (mm), *D_i_* (mm) and *D_k_* (mm), and calculated values of Modified Wentworth Roundness, Maximum Projection Sphericity and Oblate–Prolate Index for 148 particles.
8	Station 5	Measurements of *a*-axis (mm), *b*-axis (mm), *c*-axis (mm), *D_i_* (mm) and *D_k_* (mm), and calculated values of Modified Wentworth Roundness, Maximum Projection Sphericity and Oblate–Prolate Index for 126 particles.
9	Station 6	Measurements of *a*-axis (mm), *b*-axis (mm), *c*-axis (mm), *D_i_* (mm) and *D_k_* (mm), and calculated values of Modified Wentworth Roundness, Maximum Projection Sphericity and Oblate–Prolate Index for 123 particles.
10	Station 7	Measurements of *a*-axis (mm), *b*-axis (mm), *c*-axis (mm), *D_i_* (mm) and *D_k_* (mm), and calculated values of Modified Wentworth Roundness, Maximum Projection Sphericity and Oblate–Prolate Index for 99 particles.
11	Navilawa Monzonite Density	Estimation of the density of fresh Navilawa Monzonite

**Table 2:** Location of stations on the Sabeto River.

Station	Latitude	Longitude	River distance from the downstream boundary of the Navilawa Monzonite outcrop (km)	Description
0	17°41′22.75″S	177°35′04.58″E	–2.689	Site of collection of gravels of fresh Navilawa Monzonite undergoing physical breakdown
1	17°42′03.92″S	177°35′05.80″E	–1.005	Location of cross-sectional transect for bedload sampling
2	17°42′32.48″S	177°34′48.56″E	0.050	Location of cross-sectional transect for bedload sampling
3	17°42′43.95″S	177°34′36.28″E	0.714	Location of cross-sectional transect for bedload sampling
4	17°43′00.10″S	177°34′16.15″E	1.462	Location of cross-sectional transect for bedload sampling
5	17°43′43.64″S	177°33′15.45″E	4.367	Location of cross-sectional transect for bedload sampling
6	17°43′48.30″S	177°32′40.12″E	5.508	Location of cross-sectional transect for bedload sampling
7	17°43′05.85″S	177°31′20.31″E	8.755	Location of cross-sectional transect for bedload sampling

**Table 3:** Summary of measured data in file "SabetoRiverMonzoniteBedloadShapeDataset.xlsx", Mendeley Data [1].

Station	Number of clasts	*a*-axis range (mm)	*b*-axis range (mm)	*c*-axis range (mm)	*D_i_* range (mm)	*D_k_* range (mm)	Range of mass (g)
0	23	103–249	61–212	15–122	60–170	1–1	NA
1	130	25–237	18–187	10–93	15–165	1–50	NA
2	125	18–174	13–130	8–95	14–130	2–52	4.35–2,357
3	109	19–359	14–205	10–95	12–188	2–80	2.83–9,300
4	148	17–386	12–270	7−136	12–228	2–60	1.90–12,874
5	126	22–351	16–281	9–204	16–244	4–140	4.41–20,600
6	123	18–390	15–337	7–145	12–320	4–88	3.41–21,100
7	99	34–236	20–212	16–148	18–204	4–92	14.61–8,486

**Summary**	**883**	**17–386**	**12–337**	**7–204**	**12−320**	**1–140**	**1.90–21,100**

**Table 4:** Summary of calculated data in file "SabetoRiverMonzoniteBedloadShapeDataset.xlsx", Mendeley Data [1].

Station	Number of clasts	Modified Wentworth Roundness range	Maximum Projection Sphericity range	Oblate-Prolate Index range	Range of estimated volume (cm^3^)
0	23	0.01–0.02	0.27–0.88	‒8.94–11.96	NA
1	130	0.01–0.93	0.39–0.90	‒10.44–14.45	NA
2	125	0.04–0.67	0.39–0.89	‒13.07–10.65	1.6–893
3	109	0.06–0.71	0.33–0.88	‒21.51–12.56	1.1–3,523
4	148	0.08–0.83	0.36–0.94	‒11.79–11.22	0.7–4,877
5	126	0.08–0.83	0.40–0.88	‒16.10–12.42	1.7–7,731
6	123	0.10–0.83	0.36–0.93	‒16.62–15.01	1.3–7,918
7	99	0.07–0.82	0.38–0.96	‒17.24–9.56	5.5–3,212

**Summary**	**883**	**0.01–0.93**	**0.27–0.96**	**‒21.51–14.45**	**0.7–7,918**

Table 5 describes and lists the parameters of the dataset in the worksheets for each station. The data are presented in XLSX data table format and sorted sequentially by *b*-axis, *a*-axis and *c*-axis lengths in ascending order.

**Table 5:** Station worksheet parameters and description in file "SabetoRiverMonzoniteBedloadShapeDataset.xlsx", Mendeley Data [1].

Worksheet Column	Parameter	Description
A		This column is not used
B	Line	Data line number
C	*a*-axis (mm)	Clast long-axis measurement, in mm
D	*b*-axis (mm)	Clast intermediate-axis measurement, in mm
E	*c*-axis (mm)	Clast short-axis measurement, in mm
F	*D_i_* (mm)	Measured diameter of the maximum inscribed circle of the particle, in mm
G	*D_k_* (mm)	Measured diameter of the sharpest corner on the maximum projection outline, in mm
H	Mass (g)	Measured mass of clast, in g
I	Modified Wentworth Roundness	Calculated using the formula, $Modified\;Wentworth\;Roundness=\frac{D_{k}}{D_{i}}$ The result is dimensionless.
J	Maximum Projection Sphericity	Calculated using the formula, $Maximum\;Projection\;Sphericity={\left(\frac{c^{2}}{ab}\right)}^{1/3}$ The result is dimensionless.
K	Oblate–Prolate Index	Calculated using the formula, $\mathrm{Obla}\mathrm{te}-\mathrm{Prol}\mathrm{ate}\;\mathrm{Index}=\frac{10\{[(a-b)/(a-c)]-0.5\}}{c/a}$ The result is dimensionless.
L	Estimated Volume (cm^3^)	Calculated, in cm^3,^ using the formula, $volume\;=\;\frac{mass}{density}$ The Navilawa Monzonite density is estimated to be 2640 kg m^–3^ (see the "Rock Density" worksheet)

## 2 Experimental Design, Materials and Methods

Fluvial bedload sampling was undertaken in the basin of the Sabeto River, Ba Province, western Viti Levu, Fiji (Fig. 1). The Sabeto River is an active, bedload transporting, gravel-bed system [3]. The lithology of the river gravels reflects the make-up of the rocks exposed in the catchment. The clasts include granitic rocks of the intrusive Navilawa Monzonite and conglomerates of the Nadele Breccia, the Koroyanitu Breccia and the Sabeto Volcanics [4], [5], [6]. The Navilawa Monzonite crops out in the upper reaches of the river. The river has entrenched a channel that extends approximately 2.7 km across the outcrop, resulting in the direct supply of monzonite gravels to the fluvial system. The existence of a constrained and well-defined source of a distinctive isotropic lithology allows us to monitor the downstream evolution of the shape and size of fluvial gravels from a known source without the complications that might arise from the continuous addition of new material to the system.

Station 0, located alongside the bedrock channel within the outcrop of the Navilawa Monzonite, was selected to provide information on the shape of the bedload material prior to its introduction to the fluvial system. The sampled clasts were the product of the active physical breakdown of fresh Navilawa Monzonite. The availability of this material gives us the opportunity to observe the nature of the precursor particles as they are liberated from the rock and before they experience fluvial modification.

Sampling of bedload gravels of Navilawa Monzonite was carried out from above the downstream boundary of the outcrop of the monzonite over a river distance of some 9.7 km (Fig. 1). Seven stations (1–7) were established along this reach. Station 1 was located above the downstream boundary of the outcrop of the Navilawa Monzonite, with Stations 2–7 located progressively further downstream of the outcrop. The downstream limit of sampling lies at the confluence of Nawainiu Creek with the trunk stream. The Navilawa Monzonite outcrops along the headwaters of this creek and may contribute new sources of monzonite to the system, potentially complicating the story of particle evolution.

Sampling of the fluvial bedload was carried out in the active stream channel (defined as comprising the unvegetated part of the cross-section). At each station, a transect was established normal to the river and across the entire width of the active channel. Every gravel of Navilawa Monzonite ≳ 10 mm lying on the stream bed beneath the transect line was sampled. If a minimum of 99 clasts was not obtained, the transect was moved 1 m downstream and sampling continued along the entire length of the new transect. At Station 0, by contrast, the entire volume of physically loosened particles was collected from the outcrop for measurement.

Samples were taken back to the laboratory where length and mass were determined. Following the procedures of Gale and Hoare (118–122) [7], the long (*a*), intermediate (*b*) and short (*c*) axis of each clast was measured, along with the diameter of the sharpest corner of the maximum projection outline (*D_i_*) and the diameter of the maximum inscribed circle (*D_k_*). Note that our measurement of particle axial length followed the protocol of Krumbein (65,66) [8], in which the *b*-axis represents the longest axis orthogonal to the longest (*a*) axis and the *c*-axis represents the longest axis orthogonal to the *a*–*b* plane. Using this information, the Modified Wentworth Roundness [9], Maximum Projection Sphericity [10,11] and Oblate–Prolate Index [9] of each particle were calculated.

Five rectilinear blocks of fresh Navilawa Monzonite of varying sizes were sawn and their volume and mass determined in order to calculate the density of the monzonite.

## Ethics Statement

The work did not involve the use of human subjects, did not involve animal experiments and did not involve the collection of data from social media platforms.

## CRediT authorship contribution statement

**Zelina Z. Ibrahim:** Conceptualization, Investigation, Data curation, Validation, Visualization, Writing – original draft. **S.J. Gale:** Conceptualization, Methodology, Project administration, Supervision, Investigation, Writing – review & editing.

## Declaration of Competing Interest

The authors declare that they have no known competing financial interests or personal relationships that have or could be perceived to have influenced the work reported in this article.

## Data Availability

Measurements of river bed gravel samples from the Sabeto River, Fiji (Original data) (Mendeley Data).

## References

[1] Ibrahim Z.Z., Gale S.J. (2021). Measurements of river bed gravel samples from the Sabeto River, Fiji. Mendeley Data.

[2] Department of Lands and Surveys, (1985). Fiji map series 31, Topographic map 1:50000, Sheet L27 Lautoka.

[3] Gale S.J., Ibrahim Z.Z., Lal J., Sicinilawa U.B.T. (2019). Downstream fining in a megaclast-dominated fluvial system: the Sabeto River of western Viti Levu, Fiji. Geomorphology.

[4] Rao B. (1983).

[5] Hathway B. (1993). The Nadi Basin: Neogene strike-slip faulting and sedimentation in a fragmented arc, western Viti Levu, Fiji. J. Geol. Soc. Lond..

[6] Rodda P. (2022).

[7] Gale S.J., Hoare P.G. (2011).

[8] Krumbein W.C. (1941). Measurement and geological significance of shape and roundness of sedimentary particles. J. Sediment. Petrol..

[9] Dobkins J.E., Folk R.L. (1970). Shape development on Tahiti-Nui. J. Sediment. Petrol..

[10] Folk R.L. (1955). Student operator error in determination of roundness, sphericity, and grain size. J. Sediment. Petrol..

[11] Sneed E.D., Folk R.L. (1958). Pebbles in the lower Colorado river, Texas a study in particle morphogenesis. J. Geol..

